# Growth Hormone Deficiency Following Traumatic Brain Injury

**DOI:** 10.3390/ijms20133323

**Published:** 2019-07-06

**Authors:** Oratile Kgosidialwa, Osamah Hakami, Hafiz Muhammad Zia-Ul-Hussnain, Amar Agha

**Affiliations:** Academic Department of Endocrinology, Beaumont Hospital, Royal College of Surgeons, Dublin D09V2N0, Ireland

**Keywords:** traumatic brain injury, growth hormone deficiency, hypopituitarism

## Abstract

Traumatic brain injury (TBI) is fairly common and annually affects millions of people worldwide. Post traumatic hypopituitarism (PTHP) has been increasingly recognized as an important and prevalent clinical entity. Growth hormone deficiency (GHD) is the most common pituitary hormone deficit in long-term survivors of TBI. The pathophysiology of GHD post TBI is thought to be multifactorial including primary and secondary mechanisms. An interplay of ischemia, cytotoxicity, and inflammation post TBI have been suggested, resulting in pituitary hormone deficits. Signs and symptoms of GHD can overlap with those of TBI and may delay rehabilitation/recovery if not recognized and treated. Screening for GHD is recommended in the chronic phase, at least six months to a year after TBI as GH may recover in those with GHD in the acute phase; conversely, it may manifest in those with a previously intact GH axis. Dynamic testing is the standard method to diagnose GHD in this population. GHD is associated with long-term poor medical outcomes. Treatment with recombinant human growth hormone (rhGH) seems to ameliorate some of these features. This review will discuss the frequency and pathophysiology of GHD post TBI, its clinical consequences, and the outcomes of treatment with GH replacement.

## 1. Introduction

Traumatic brain injury (TBI) is defined as non-degenerative, non-congenital insult to the brain from an external mechanical force causing temporary or permanent neurological dysfunction, which may result in the impairment of cognitive, physical, and psychosocial functions [1]. TBI can be classified according to the mechanism of injury (open versus closed). The clinical severity is commonly assessed according to the Glasgow Coma Scale (GCS) or injury severity score, and structurally by imaging and prognostic models [2]. Historically, GCS has evolved as the universal classification of TBI severity with GCS scores of 13 to 15 classified as mild, 9 to 12 as moderate, and 3 to 8 as severe TBI [3]. A recent study found that the incidence of TBI was estimated to be 69 million (95% CI 64–74 million) worldwide [4]. There exist differences in the incidence of TBI across the world with low- and middle-income countries experiencing nearly three times more cases of TBI proportionally than high income countries [4]. Complications of TBI include increased mortality and morbidity.

Post traumatic hypopituitarism (PTHP), a recognized clinical entity for a century, is one contributor to morbidity in this cohort [5]. This was previously thought to be rare, but in the last 15 years, it has received more recognition as a common complication of TBI. Hypopituitarism is defined as a deficiency in the production of one, several, or all of the pituitary hormones, regardless of the cause. This is of clinical importance as unrecognized PTHP can impair rehabilitation and recovery [6]. PTHP is common, with the prevalence of PTHP for at least one pituitary hormone estimated at 28% [7]. Severe TBI seems to confer the highest risk of PTHP [7]. In this article, we reviewed growth hormone deficiency (GHD) following moderate/severe TBI. 

## 2. Prevalence 

The reported prevalence of GHD after TBI is highly variable (Table 1 and Table 2). This variability in prevalence is possibly due to a number of factors including the timing of the assessment, injury severity, age of onset, and the methods used to diagnose/confirm pituitary hormone dysfunction [6]. The prevalence of acute GHD, within one month of TBI, has been reported as between 2–30% [8,9,10] (Table 1). In the acute TBI setting, methods of assessment include basal IGF-1 and growth hormone measurement as well as glucagon stimulation test. Unfortunately, random GH and basal IGF-1 values are not a reliable measure of GHD. 

In the majority of studies, GHD is the most common anterior pituitary hormone deficiency in the chronic phase of TBI and ranges between 10–63.6% [9,11,12,13,14,15,16,17,18,19] (Table 2). A lower incidence was reported when using a strict diagnostic criterion. The study that reported the highest incidence included both partial and severe GHD [13]. This review will discuss GHD diagnosed in the chronic phase post TBI as this is deemed to be clinically relevant, especially in the rehabilitative period. 

## 3. GH/IGF-1 and the Brain 

Growth hormone (GH) is a peptide hormone synthesized by somatotropic cells of the anterior pituitary. Its release is regulated primarily by hypothalamic peptides and negative feedback. GH releasing hormone (GHRH) stimulates GH release, whereas somatostatin inhibits its release. GH acts via two independent mechanisms: directly via GH receptors (GHR) and by inducing the secretion of insulin growth factor 1 (IGF-1) in the liver. GHR is a transmembrane receptor found on the cell surface of most cells. Centrally, GHR is expressed in high concentrations in the choroid plexus, hippocampus, hypothalamus, and the pituitary [20,21]. The choroid plexus, found in the ventricles of the brain, is made up of modified ependymal cells [22]. Its main function is to release cerebrospinal fluid (CSF) and also forms the blood–CSF barrier via tight junctions between adjacent epithelial cells. GH is thought to cross the blood brain barrier (BBB) via the receptor-mediated transport in the choroid plexus [23]. The hippocampus is part of the limbic system and is involved in memory, learning, and emotions. Thus, the cognition and quality of life problems experienced by patients with GHD may be explained by the reduced expression of GH activity in these areas of the brain. Peripherally, GHR is found in many other tissues including the liver, muscle, bone, and adipose [24].

GH is a pleiotropic hormone and is one of the major players of the nervous system development. It also promotes cell growth and differentiation [25]. GH has been shown to play an important role in neuroprotection and neuro-regeneration [26,27,28]. It has also been shown to be one of the key hormones involved in the regulation of appetite, cognitive function, energy, memory, mood, neuroprotection, sleep, and well-being [23]. Peripherally, GH is an anabolic hormone, known to increase growth in skeletal and soft tissue [29]. It also plays an important role in metabolism. 

GH binding to the GHR in target tissue stimulates the production and secretion of IGF-1 from many tissues, particularly the liver [30]. However, some IGF-1 is also produced locally by brain tissue. IGF-1 is a single polypeptide chain of 70 amino acids with 43% homology to proinsulin [31]. It exerts its physiologic activity by binding to the IGF-1 receptor (IGF-1R), a glycoprotein. Some IGF-1 is produced locally in the brain, but like GH, also crosses the BBB via transport mediated uptake [32]. IGF-1 and its receptors have also been shown to be present in the adult brain and to be involved in the pathogenesis of several growth-related neurological disorders [33]. Indeed, low IGF-1 levels have been linked to cognitive impairment [34]. 

The GH/IGF-1 axis is important for central nervous system tissue growth, development, myelination, and plasticity [35]. In rat studies, GH has been shown to stimulate neuronal proliferation and differentiation and improve cognitive function [36,37]. It has been shown to be neuroprotective in hypoxic/ischemic injury partly via its anti-apoptotic effect [38]. In rat studies, IGF-I seems to be emerging as a restorative molecule for increasing hippocampal neurogenesis and memory accuracy in aged individuals [39]. It is known that impaired release of GH/IGF-1 such as that seen with advancing age leads to severe alterations in brain structures and functions [40].

Outside the CNS, the GH/IGF-1 axis is important for other functions. These include stimulating lipolysis, reducing hepatic triglyceride secretion, activating the nitric oxide system (and reducing vascular tone), increasing cardiac performance and exercise capacity, and promoting longitudinal skeletal growth [29]. 

## 4. Pathophysiology of GHD after TBI 

Multiple theories have been described to explain the pathophysiology of GHD post TBI. The most widely accepted theory is that of ischemic injury to the pituitary [41,42]. Acute TBI is characterized by two injury phases: primary and secondary [43]. In the primary phase, direct trauma to the brain at the time of the initial impact results in a series of biochemical processes that result in secondary brain injury [43]. Primary brain injury may lead to pituitary stalk traumatic transection, direct trauma to the hypothalamus and pituitary, or the compressive effect of increased intracranial pressure, resulting in ischemia and necrosis of the anterior pituitary and thus hypopituitarism [44,45]. The pituitary stalk that connects the hypothalamus to the pituitary gland is structurally fragile and vulnerable to the effects of TBI [46]. The anterior pituitary does not have direct arterial blood-supply, but instead gets all of its blood supply via the hypophyseal portal vessels [47]. The long hypophyseal portal veins connect the hypothalamus to the anterior pituitary providing 70–90% of the anterior pituitary blood supply, whereas the shorter portal vessels originating in the lower part of the pituitary stalk and the posterior lobe provide the remaining 10–30% [42,48]. The somatotropic cells are located laterally in the pituitary with the majority of its vascular supply provided by the long portal veins that have an anterolateral distribution in the gland [49]. GH releasing hormone (GHRH) neurons in the hypothalamus also seem to be vulnerable to ischemic injury due to their position [50]. 

Contributing to the initial brain injury, other factors associated with trauma such as hypotension and hypoxia may cause ischemic injury to the pituitary at this time. To support the theory of vascular injury/ ischemia as a cause of PTHP, magnetic resonance imaging (MRI) in the acute phase has shown swelling of the pituitary gland compared to healthy controls, whereas in the chronic phase, volume loss or empty sella has been described in patients who went on to develop PTHP [51,52]. 

### 4.1. Molecular Mechanisms of the Growth Hormone Deficiency after Traumatic Brain Injury

**Figure 1:**
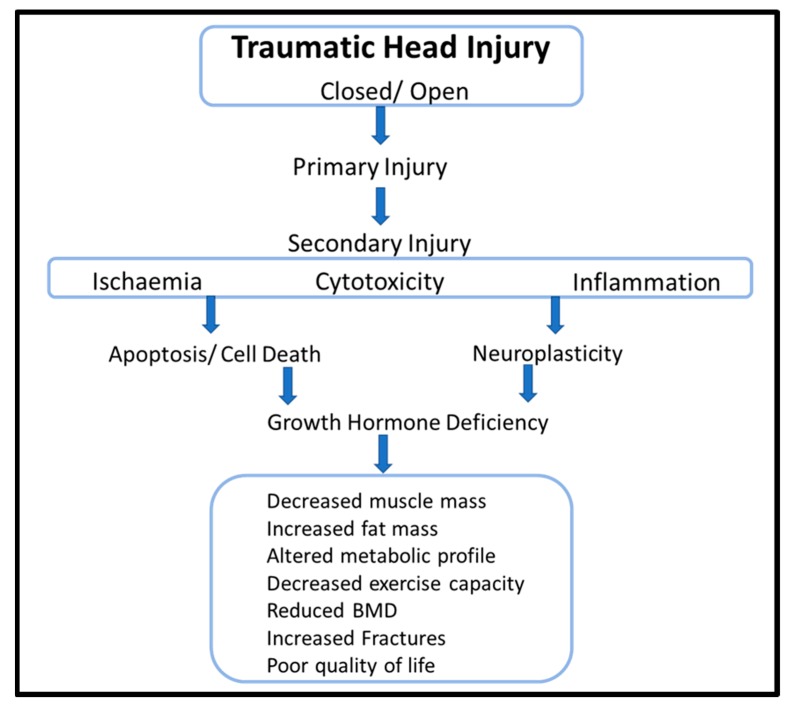
Pathophysiology and clinical features of growth hormone deficiency following traumatic brain injury.

After the initial primary phase of TBI, the secondary phase is characterized by a combination of ischemic, cytotoxic, and inflammatory processes that further propagate the brain injury (Figure 1) [43]. As described below, neuroinflammation is strongly implicated in the molecular pathophysiology of PTHP and thus GHD. 

#### 4.1.1. Ischemia

It is hypothesized that the initial hypoxic-ischemic insult that occurs at the time of trauma leads to subsequent oxidative stress and cytotoxicity leading to the death of neuronal cells by apoptosis or necrosis [53]. Histological examination of patients post-TBI showed that the underlying pituitary pathology in patients dying after TBI were acute infarction of the pituitary, capsular hemorrhage around the pituitary, anterior lobe necrosis, and stalk necrosis [44,45,54]. 

#### 4.1.2. Cytotoxicity

Secondary ischemic brain injury, focal contusions, sustained high intracranial pressure, and poor outcome have been shown to be strongly associated with high excitatory amino acid levels (glutamate) in patients with TBI [55]. At the time of the TBI, there is a release of excitotoxins such as glutamate and aspartate that act on the N-methyl-D-aspartate (NMDA) channel, altering cell wall permeability with an uncontrolled shift of sodium, potassium, calcium, and activation of calcineurin and calmodulin [43]. This ultimately leads to severe cell swelling and cell death [55]. 

#### 4.1.3. Inflammation

Cortical brain injury might induce pathological changes in structures distal to the cortical injury like the hypothalamus and pituitary gland by persistence and spread of inflammatory factors at the site of injury, resulting in secondary necrosis and apoptosis of distal brain tissue [56]. Rat models have shown that pro inflammatory cytokines such as interleukin 1 (IL-1) and tumor necrosis factor (TNF), released as a result of TBI at the primary injury site of injury, may also contribute to the development of PTHP [57]. Rat models have also shown a significant increase in the expression of IL-1β and glial fibrillary acidic protein (GFAP) in the hypothalamus and pituitary post bilateral cortical brain injury [56]. It is hypothesized that the inflammatory factors produced in the cortex diffuse to distant sites through the ventricles or by movement through extracellular fluid and spaces, activating further cytokine (IL-1) production downstream from the initial injury and activating a rolling cascade of inflammatory reactions [56,58]. 

#### 4.1.4. Other Possible Mechanisms 

There is also some evidence to suggest that autoimmunity is a contributor to pituitary hormonal deficits post TBI. Anti-pituitary antibodies (APA) have been detected in patients with TBI when compared to normal controls [59]. Tanriverdi et al. found a positive correlation between APA positivity and PTHP, with close to 50% of the patients with positive antibodies developing hypopituitarism three years after TBI [59]. In the same study, the authors found that high APA titers were associated with a low GH response to the GH releasing hormone (GHRH) + GH related peptide (GHRP)-6 test. When these patients were followed up for a period of five years, those with pituitary dysfunction had significantly higher titers of both anti-hypothalamus antibodies (AHA) and APA [60]. In another study by the same group, AHA and not APA was significantly correlated with the development of PTHP in a cohort of boxers [61]. However, these autoantibodies were non-specific and have been detected in other forms of pituitary pathology such as Sheehan’s syndrome and sometimes in patients without any pituitary/hypothalamus pathology [62,63]. Thus, no causal relationship can be concluded between GHD and autoimmunity in the context of TBI. 

Genetic predisposition to the development of PTHP has also been implicated. Apolipoprotein E (APOE) is the major apolipoprotein produced in the central nervous system. It is synthesized by astrocytes, microglia, and neurons under conditions of stress and has an inhibitory effect on the neuroinflammatory cascade following injury [53,64]. Predominantly, patients with the APOE ε3/ε3 genotype seem to have a lower risk of developing PTHP than patients with other genotypes [65].

## 5. Signs and Symptoms 

In adults, the signs and symptoms of GHD can be subtle and are shown in Table 3. There is some overlap between the symptoms of GHD and those from TBI, which may contribute to delays in the diagnosis of GHD post TBI. GHD, regardless of cause, is associated with poor quality of life (QoL), diminished lean body mass (LBM), increased body fat, disrupted lipoprotein and carbohydrate metabolism, reduced bone mineral density, and impaired cardiac function [66,67]. These may be partially ameliorated by treatment with recombinant human GH (rhGH) replacement. The literature is more robust for growth hormone treatment improving cognition and QoL, and not for all the other parameters as discussed below. 

## 6. Mild Traumatic Brain Injury

Mild TBI (MTBI) is commonly defined on a GCS of 13 to 15 and is the most common type of head trauma. Routine screening of PTHP is, however, not routinely advised in this group as it is not cost effective and the evidence for significant pituitary dysfunction following a single MTBI is rather weak. Screening is recommended for patients with complicated MTBI, especially those with repetitive MTBI (e.g., boxing) or those with blast wave injuries from explosives such as that seen in wars [68,69], as this may be associated with an appreciable incidence of isolated GHD [68,70,71]. In addition, MTBI patients who need hospitalization for more than 24 h, intensive care monitoring, neurosurgical intervention, or anatomical changes on initial brain imaging would benefit from screening for GHD [72].

Conventional MRI frequently shows no abnormalities in patients with PTHP/GHD following MTBI. The apparent diffusion coefficient (ADC) measures the diffusion of water molecules within cellular structures and thus brain tissue integrity [73] and seems to correlate with GCS and degree of neurologic dysfunction where the MRI brain was reported as normal [74]. In one prospective study, forty-two patients admitted with MTBI with normal appearing brain imaging were scanned seven days after injury using diffusion-weighted imaging to quantify the changes in pituitary ADC [75]. Mean pituitary ADC values were compared with 30 healthy controls. The TBI group showed a significant decrease in pituitary ADC when compared to the controls, suggesting microstructural damage in the pituitary gland. Furthermore, the mean ADC was much lower in TBI patients with PTHP when compared to those with normal pituitary function. Therefore, pituitary ADC is a sensitive and independent marker of pituitary damage post TBI and may be particularly useful in MTBI.

## 7. Evidence for Treatment of Post-Traumatic GHD

The brain is neuroplastic with a capacity to repair itself after injury. The GH/IGF-1 axis has been shown to have a major role in neuronal repair after TBI [35]. Acutely after TBI, GH and IGF-1 expression are upregulated regardless of GH status [76,77]. However, the clinical significance of this acute upregulation is still not clear. When exogenous GH was given to rats post TBI (both GHD and GH sufficient), this seemed to increase the repair of damaged hippocampal neurons and other areas of the brain [36]. In patients with traditional causes of hypopituitarism such as pituitary tumors, GH deficiency is associated with poor metabolic, skeletal, and quality of life sequelae and increased cardiovascular (CV) risks, and treatment of adult GH deficiency has been shown to be beneficial [78]. However, in the field of posttraumatic GH deficiency, the evidence of the benefit from GH replacement is scant and discussed below. 

### 7.1. Cognition 

GHD post TBI has been associated with a variety of cognitive issues including poor verbal learning, verbal short-term memory, and attention [13,66]. GHD also seems to be associated with poor mental health outcomes. Popovic et al. showed that paranoid ideation and somatization were negatively correlated with the peak GH responses to dynamic testing [66]. One meta-analysis showed moderate to large impairments in GH deficient patients in each of the cognitive domains assessed when compared to the matched controls [79]. There is some evidence to suggest that TBI with GHD confers a worse risk for the development of poor cognition outcomes when compared to TBI with an intact GH axis. In one study, patients with GHD after TBI showed decreased cerebral glucose metabolism in cortical areas involved in the regulation of intellectual function, executive function, and working memory [80]. 

It is well established that patients with GHD from non-traumatic causes benefit from treatment with rhGH [81,82]. One observational study found that the GH peak value using GHRH + ARG (arginine) was an independent predictor of positive outcomes, indicating that recovery during an intensive rehabilitation program after TBI may be positively influenced by normal GH secretion and suggests that GH replacement may be considered in the cohort of posttraumatic GHD [83]. Patients with GHD post TBI seem to have significant improvements in cognitive rehabilitation when treated with open-labelled rhGH as assessed by the Wechsler adult intelligence scale (WAIS) [84].

In one meta-analysis assessing all patients with GHD regardless of cause, patients treated with GH replacement had moderate improvements in cognitive performance, particularly attention and memory when compared to the baseline [79]. These patients, however, still performed moderately to largely below that of the controls. There is also some evidence to suggest that stopping treatment may worsen symptoms. In one small non randomized study of six patients, Maric et al. reported the worsening of verbal and non-verbal memory in patients who stopped rhGH therapy for 12 months [85]. When compared with untreated patients, GHD patients on GH seemed to benefit more, especially those with worse symptoms prior to commencing treatment [86].

### 7.2. Metabolic and Cardiovascular 

Outside of TBI, GHD is associated with reduced LBM, muscle mass, and muscle strength. It is well known that GH and IGF-1 have anabolic actions on skeletal muscle tissue [87]. Whole-body protein turnover studies using infusions of isotopically labelled leucine have shown that adults with GHD have reduced protein synthesis when compared with healthy controls [87,88]. Patients with TBI have been found to have below normal aerobic capacity, a well-established measure of physical endurance and fatigue resistance, which may further delay or hinder the rehabilitative process [89]. Patients with TBI and GHD seem to do even worse than those without GHD. One study found that patients with TBI and a normal GH axis showed suboptimal aerobic capacity and those with GHD performed even worse [89]. There is evidence to support the use of rhGH in this cohort [90]. 

Although evidence exists for growth hormone treatment improving skeletal muscle mass in the GHD of other causes, the literature is not quite as robust for TBI patients. One study showed an improvement in the muscle mass of male and not female patients with TBI and GHD [90]. A case study of one patient showed an improvement in muscle force production, body composition, and aerobic capacity after treatment with rhGH for 12 months [91]. 

Data seem to support metabolic disturbances in patients with post traumatic GHD. A study by Klose et al. showed a high low-density lipoprotein-cholesterol (LDL), total cholesterol, waist circumference, and total fat mass in patients with post traumatic hypopituitarism, mainly GHD [92]. Treatment of these patients has shown mixed results. In one observational study, there was no change in the weight or waist to hip ratio in GHD patients post TBI treated with rhGH for a year in the KIMS database (Pfizer International Metabolic Database) [93]. Similarly, no change was observed in the non-functioning pituitary adenoma (NFPA) group treated for the same time period. In that same study, there was no difference in the lipid parameters in GHD patients treated with GH replacement. Conversely, there was some improvement in the LDL in GHD patients secondary to NFPAs after a year of treatment with rhGH [94]. A case study of two patients with GHD secondary to sports related TBI showed some improvement in lipid profile and body composition after a 6-month treatment with rhGH [61]. In another study, there was an improvement in blood pressure, total cholesterol, and LDL after 1-year treatment in patients with GHD post TBI [94]. Hypopituitary patients, especially GHD, are at increased risk of cardiovascular disease and mortality [95]. There are scant data to suggest that GH replacement in hypopituitarism may be associated with a reduced risk of myocardial infarction, but no randomized placebo-controlled studies have been conducted [96].

### 7.3. Bone 

Hypopituitary patients adequately replaced with glucocorticoids and thyroid hormones have a higher risk of osteopenia, osteoporosis, and vertebral fractures in general [97]. The prevalence of all fractures among patients in the KIMS database was 2.7 times higher than the control population [97]. Gender and age did not seem to make a difference. Observational studies suggest that GH replacement increases BMD [98,99] and may mitigate the increased fracture risk associated with GHD [97], but specific data for skeletal outcomes in TBI induced GHD are lacking. 

### 7.4. Quality of Life (QoL)

GHD regardless of cause is associated with poor QoL [100,101]. Patients with GHD due to TBI are more likely to be depressed and report a poor quality of life [82,102]. Poor QoL is primarily in the domains of physical health, energy and fatigue, emotional well-being, pain, and general health [92,102]. This perceived poor QoL would negatively impact on recovery and rehabilitation after TBI. Interestingly, patients with GHD secondary to TBI when compared to those with GHD secondary to NFPA, biochemically seemed to have less severe GHD, but worse QoL scores [94]. In general, treatment of GHD due to any cause seems to improve QoL as measured by the QoL-AGHDA (Quality of Life Assessment of Growth Hormone Deficiency in Adults) score and other instruments [103]. The QoL-AGHDA score was introduced to measure the impact of GH replacement on patients over time [104]. When compared with patients with GHD due to NFPA, patients with TBI seemed to have a better outcome in terms of QoL especially in the domains of socialization, self-confidence, and tenseness [94]. This improvement was sustained over the long term, up to eight years. This sustained improvement, however, is based on continuation of treatment. 

## 8. Who and When to Test? 

PTHP and GHD are often underdiagnosed in clinical practice [105]. Even with much publicized work on PTHP, one study found that patients with GHD post TBI were diagnosed on average two and a half years later after the primary onset of disease when compared to those with NFPA [94]. Delayed diagnosis of GHD post TBI may contribute to poor outcomes as described previously and hinder rehabilitation and recovery. Patients with severe GHD post TBI have been shown to have a delayed admission to post-acute rehabilitation centers [6]. 

Severe TBI as defined by the GCS scale seems to confer the highest risk of developing PTHP including GHD [7,14,106,107]. Better quality data are available regarding the risk of PTHP after moderate and severe TBI compared to mild TBI; hence the former group should be the target for routine screening [108,109]. Mild complicated TBI, defined as a need for hospitalization for more than 24 h, need for ICU monitoring, and/or neurosurgical intervention and any anatomical changes on initial brain imaging, would also justify screening [72]. 

Plasma insulin growth factor 1 (IGF-1) levels do not reliably reflect GH secretion or action in acute illness [10]. In addition, approximately 50% of patients with chronic GHD will exhibit a normal IGF-1 level [110]. Thus, the plasma IGF-1 level lacks sensitivity to diagnose GHD post TBI and as a result cannot be used as a screening tool in these patients [6,111]. Indeed, in patients with TBI, there was no correlation between plasma IGF-1 levels and adverse sequelae associated with the GH deficiency such as BMI-adjusted LDL, total cholesterol, waist circumference, and total fat mass [92].

**Figure 2:**
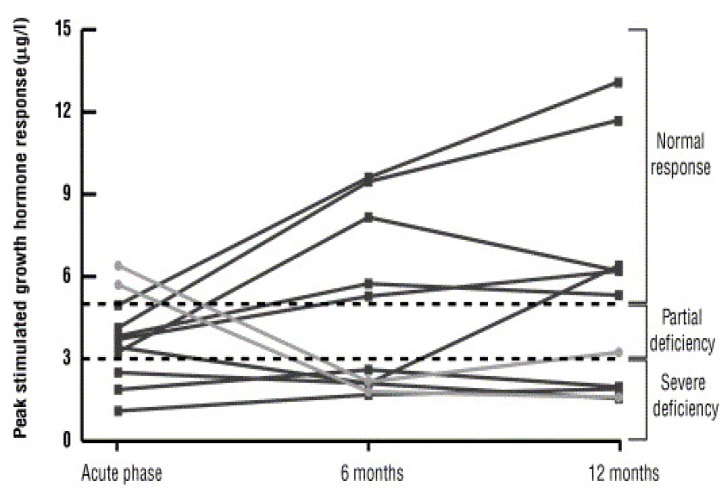
Peak growth hormone (GH) responses to glucagon stimulation in patients with early and late growth hormone deficiencies showing recovery of GH secretion in some patients in the post-acute phase, while others developed new deficiencies later in the chronic phase of TBI. Normal response is GH above 5 mcg/l. Image from senior author’s own study; Reference [112].

Dynamic testing is therefore recommended in the chronic phase at least six months after the initial TBI because hypopituitarism can occur early following TBI and may recover spontaneously in some patients in the post-acute phase. Conversely, new pituitary hormone abnormalities can occur later on and persist (Figure 2) [112]. The GH research society guidelines recommend that patients with three or more pituitary hormone deficits and an IGF-I level below the reference range do not require dynamic testing as they have >97% chance of being GH deficient [82]. However, patients with TBI often have isolated pituitary deficits or partial hypopituitarism and thus require dynamic testing [1]. Dynamic testing using the insulin tolerance test (ITT), growth hormone releasing hormone (GHRH) + arginine, GHRH + GH releasing peptide-6, glucagon stimulation test (GST) are acceptable tests for assessing growth hormone reserve and deficiency. The choice of the test depends on patient factors, the availability of the secretagogue, and physician/center preference. Various centers use the different dynamic tests as shown in Table 2. In addition, the different methods of diagnosing GHD have different cut offs. The ITT is considered the gold standard for assessing GHD. However, this test is contraindicated in patients with a prior history of cardiac disease and seizures. Given that a significant proportion of patients with TBI are also at an increased risk of developing seizure disorders (up to 22%), the ITT is often deemed not safe in these patients, given the risk of precipitating a seizure [113]. The GHRH + arginine test is considered an alternative to the ITT. However, the lack of availability of GHRH makes the GHRH + arginine and GHRH + GHRP-6 tests difficult to carry out [114]. The GST is a suitable alternative to the ITT although unfortunately, normative cut-offs are less defined for diagnosing GHD with the GST. Ideally, these cut-offs should be established locally.

## 9. Conclusions 

GHD is the most common pituitary hormone deficiency after TBI. After the initial primary injury, secondary mechanisms that involve an interplay of ischemia, inflammation, and cytotoxicity seem to result in GHD. Posttraumatic GHD is associated with adverse sequelae, which may impair recovery and rehabilitation. The poor outcomes that are seen with long standing GHD in this population can be improved by treatment with rhGH. Research into treatments aimed at halting or ameliorating the secondary phase of TBI may be helpful in preserving the function of the anterior pituitary in patients post TBI.

## Figures and Tables

**Table 1:** Prevalence of growth hormone deficiency occurring within one month of traumatic brain injury.

Study	Number of Participants	Severity (GCS)	Median Age at TBI (Range) (Years)	Timing of Testing Post TBI (Days)	GHD (%)
Olivecrona et al. [8]	45	≤8	15–64	1 4	30 2
Tanriverdi et al. [9]	52	3–15	35 (17–65)	0–1	20
Agha et al. [10]	50	8–13	37 (15–65)	7–20	18

GCS—Glasgow coma scale; TBI—Traumatic brain injury; GHD—Growth hormone deficiency.

**Table 2:** Sample studies on the prevalence of growth hormone deficiency occurring in the chronic phase post traumatic brain injury.

Study	Number of participants	Severity (GCS)	Test Used to Diagnose GHD	Median Age at TBI (Range) (Years)	Timing of Testing Post TBI (Months)	GHD (%)
Tanriverdi et al. [9]	52	3–15	GHRH + GHRP-6	35 (17–65)	12	37.7
Agha et al. [11]	102	3–13	ITT Or GHRH test + Arginine	28 (15–65)	6–36	10.7
Aimaretti et al. [12]	70	3–15	GHRH + arginine test	39	3	38.5
					12	38.6
Kozlowski et al. [13]	55	3–15	-	36.1	>12	63.6
Klose et al. [14]	104	3–15	ITT Or GHRH test + Arginine	41 (18–64)	13 (10–27)	15
Abadi et al. [15]	75	9–13	IGF-1	38 (15–54)	3	24
					6	9.3
Bondanelli et al. [16]	50	3–15	GHRH + arginine test	37.6 (20–87)	12–64	28
Hannon et al. [17]	32	<14	ITT Or GST	-	6–24	18.8
Krahulik et al. [18]	186	3–14	GHRH test + Arginine Or GST	36 (18–65)	12	13.5
Schneider et al. [19]	78	3–15	GHRH test + Arginine	36	12	10

GCS—Glasgow coma scale; TBI—Traumatic brain injury; GHD—Growth hormone deficiency; GHRH—Growth hormone releasing hormone; ITT—Insulin tolerance test; GST—Glucagon stimulation test; GHRP—Growth hormone releasing peptide.

**Table 3:** Signs and symptoms of growth hormone deficiency.

Deficient Hormone	Symptoms	Signs
GH	Poor QoL Decreased energy Low mood	Decreased muscle mass Increased fat mass Altered metabolic profile Decreased exercise capacity Reduced BMD Increased Fractures

GH—Growth Hormone; QoL—Quality of Life; BMD—Body mineral density.
